# Severity of local inflammation does not impact development of fibrosis in mouse models of intestinal fibrosis

**DOI:** 10.1038/s41598-018-33452-5

**Published:** 2018-10-12

**Authors:** A. Hünerwadel, S. Fagagnini, G. Rogler, C. Lutz, S. U. Jaeger, C. Mamie, B. Weder, P. A. Ruiz, M. Hausmann

**Affiliations:** 1 Department of Gastroenterology and Hepatology, University of Zurich, Zurich, Switzerland; 2 Dr. Margarete Fischer-Bosch Institute of Clinical Pharmacology, Stuttgart, Germany; 3 University of Tübingen, Tübingen, Germany

## Abstract

Intestinal fibrosis is thought to be a consequence of excessive tissue repair, and constitutes a common problem in patients with Crohn’s disease (CD). While fibrosis seems to require inflammation as a prerequisite it is unclear whether the severity or persistence of inflammation influences the degree of fibrosis. Our aim was to investigate the role of sustained inflammation in fibrogenesis. For the initiation of fibrosis *in vivo* the models of *Il10*^−/−^ spontaneous colitis, dextran sodium sulfate (DSS)-induced chronic colitis and heterotopic transplantation were used. In *Il10*^*−/−*^ mice, we determined a positive correlation between expression of pro-inflammatory factors (*Il1β*, *Tnf*, *Ifnγ*, *Mcp1* and *Il6*). We also found a positive correlation between the expression of pro-fibrotic factors (*Col3a1 Col1a1*, *Tgfβ* and *αSma*). In contrast, no significant correlation was determined between the expression of pro-inflammatory *Tnf* and pro-fibrotic *αSma*, *Col1a1*, *Col3a1*, collagen layer thickness and the hydroxyproline (HYP) content. Results from the DSS-induced chronic colitis model confirmed this finding. In the transplantation model for intestinal fibrosis a pronounced increase in *Mcp1*, *inos* and *Il6* in *Il10*^−/−^ as compared to WT grafts was observed, indicating more severe inflammation in *Il10*^−/−^ grafts. However, the increase of collagen over time was virtually identical in both *Il10*^−/−^ and WT grafts. Severity of inflammation during onset of fibrogenesis did not correlate with collagen deposition. Although inflammation might be a pre-requisite for the initiation of fibrosis our data suggest that it has a minor impact on the progression of fibrosis. Our results suggest that development of fibrosis and inflammation may be disconnected. This may be important for explaining the inefficacy of anti-inflammatory treatments agents in most cases of fibrotic inflammatory bowel diseases (IBD).

## Introduction

IBD, with its two entities CD and ulcerative colitis (UC), are chronic auto-inflammatory disorders with a polygenic background and multiple environmental triggers^1^. In the pathogenesis of IBD, inflammation is sustained by an exaggerated response of T-lymphocytes^2^ associated with an infiltrate of immune cells, which may cause severe local tissue damage^3^. In CD patients, the inflammation has been associated with an excessive T helper type 1 (T_H_1) and T helper type 17 (T_H_17) response with massive production of pro-inflammatory cytokines such as interleukin (IL)-1, -2, -6, -12 and interferon (IFN)-γ – at least in mouse models^4,5^.

In the intestinal mucosa, severe inflammation is followed by loss of epithelial cells and the degradation of collagen-rich extracellular matrix (ECM) in the lamina propria, leading to ulcerations. This, in turn, leads to the initiation of a reparatory process, as rapid and adequate healing is essential to restore a tight barrier and reduce the exposure of the underlying tissue to luminal antigens^3,6,7^. Excessive ECM deposition impairs gastrointestinal function due to motility abnormalities and increased wall stiffness, and promotes fibrosis, which is increasingly recognised as an important cause of morbidity and mortality in IBD. CD has a fibrostenosis phenotype at diagnosis in at least 10% of patients^8–10^, constituting a common clinical problem. Intestinal fibrosis leads to stricture formation in 30–50% of patients with CD^9,11^, and requires surgery in approximately 80% of these patients^9^. UC has been long considered a non-fibrotic disease, but some degree of submucosal fibrosis is found in almost all of colectomy specimens from patients with UC^8,12,13^, and the degree of fibrosis is proportional to the degree of chronic but not active inflammation^13^.

It is evident from clinical observations that fibrosis only develops in segments of the gut where inflammation is or was present^8^. Fibrosis occurring in gut segments that never showed signs of inflammation has not been reported. Nevertheless, the exact factors and mechanisms triggering fibrosis remain unclear. While fibrosis seems to require inflammation as a prerequisite, the mechanisms involved in the progression of fibrogenesis may be distinct and idependent of the degree of inflammation. There is evidence that intestinal fibrogenesis - once initiated – may become an independent and “self-perpetuating” process^14^.

Based on data from experiments with bleomycin (BLM) induced fibroinflammatory responses in murine lung, the association between inflammation and fibrosis is discussed since decades^15–17^, as pulmonary fibrosis was suggested to be independent from preceding inflammation.

IL-10 is considered to be one of the most important anti-inflammatory cytokines in humans, produced by various cells of the innate and adaptive immune system such as CD4^+^ T-cells, CD8^+^ T-cells, macrophages, dendritic cells (DC) and mast cells^4^. IL-10 initiates a negative regulatory pathway triggered by the production of pro-inflammatory cytokines^4,18^. IL-10 binding to its receptor (IL-10R) leads to phosphorylation, dimerization and nuclear translocation of STAT3. This leads to an increase in anti-inflammatory and a decrease in pro-inflammatory cytokine expression^4,19^ and MHC-II class molecule expression in DC and macrophages^4,18^. Together with its participation in a negative feedback loop circuit, IL-10 also plays a crucial role in the control of inflammation via CD4^+^CD25^+^ regulatory T cells (Tregs). CD4^+^CD25^+^ Tregs cells produce and require IL-10 to suppress T_H_1 and T_H_17 to control and terminate the immune response^20^. Due to their ability to conduct self-tolerance and establish an immune homeostasis, Tregs play a crucial role in the orchestration of immune responses^21^. Tregs mainly act via the inhibition of function, proliferation and cytokine production of other immune cells^21^.

Mutations in IL-10 and IL-10R are associated with a severe course of IBD^19,22,23^. Genetic variants of IL-10 in humans are associated with increased susceptibility to UC^24^. In CD patients, mutations in IL-10 protein reduce its release from cells^25^, and a mutation (3020insC) in the intracellular sensor molecule nucleotide-binding oligomerization domain containing 2 (NOD2), previously associated with CD, inhibits the ribonucleoprotein hnRNP-A1, and thereby actively blocks transcription of IL-10^19,26^. However, there are no data on the direct role of IL-10 and IL-10R in the pathomechanism of intestinal fibrosis. Nevertheless, IL-10 is known to decrease tissue remodeling protease matrix metalloproteinase (*Mmp*)9 at a transcriptional level. MMP9 is a endopeptidase responsible for the degradation of denatured collagens and basement membranes^27^ causing further tissue damage and inflammation^28,29^. MMP9 is also involved in many developmental processes, including wound healing and ECM degradation, cell migration and invasion. Recent data indicate that MMP9 plays a role in the modulation of intestinal fibrosis, and suggest that selective MMP9 inhibition is a promising therapeutic strategy for treatment of penetrating CD^30^.

Our aim was to investigate the role of sustained intestinal inflammation on fibrogenesis, and whether increased inflammation correlates with an increase in fibrotic processes. In this study, we describe the impact of inflammation on fibrogenic processes using three different models of intestinal fibrosis. Our data suggest that inflammation might be a pre-requisite for the initiation of fibrosis but has only a minor or no impact on the progression of fibrosis. This finding may help to explain the little impact most anti-inflammatory treatments have on IBD-associated fibrosis.

## Materials and Methods

### Animals

Chronic colitis was induced as described previously^31^. Mice weighing 20 g on average were bred locally in the animal facility of the University Hospital Zurich. The animals received standard laboratory mouse food and water *ad libitum*. They were housed under specific pathogen-free conditions in individually ventilated cages. During a cycle of chronic colitis mice received either 1.75% dextran sodium sulfate (DSS) in drinking water or drinking water alone over seven days. DSS was administred in a total of 3 cycles with 14 days of recovery between each cycle. Three weeks after the last DSS administration cycle the mice were euthanized.

For the model of spontaneous colitis *Il10*^−/−^ mice (C57BL/6) were used. Mice were observed until they reached 11–20 weeks of age. For the heterotopic transplantation model, male B.6129P2IL-10^tm1Cgn^/J (IL-10^−/−^) and C57BL/6-Tg (UBC-green fluorescence protein [GFP] 30Scha/J, WT) were used. Surgeries were always performed during the light cycle. The use of C57BL/6-Tg allows to determine the origin of lumen-obstructing cells or the progression of lymphocyte influx. We did not apply this option in this work, hence C57BL/6-Tg are referred as WT in the following text.

### Heterotopic intestinal transplant model

The heterotopic mouse intestinal transplant model is an adaption of the heterotopic transplantation model of intestinal fibrosis in rats, which has been previously described in detail^32^. Briefly, donor small bowel resections were extracted and transplanted subcutaneously into the neck of recipient animals. Donor small bowel proximal to the caecum was excised and flushed with 5 ml of 0.9% NaCl to remove stool, and divided into 10 mm parts. A small bowel resection was implanted into a subcutaneous pouch, and a single dose of cefazolin (Kefzol^®^, 1 g diluted in 2.5 ml aqua dist.) was applied i.p. as infection prophylaxis.

IL-10^−/−^ and WT mice were used as both donors and recipients for isogeneic transplantation. Body weight remained unchanged. 112 transplants were performed and all but 5 grafts were recovered. Grafts were explanted up to 21 days after transplantation. To compare grafts with non-transplanted tissue, 21 small bowel resections were extracted from IL-10^−/−^ mice and 19 small bowel resections were extracted from WT mice.

### qPCR

qPCR was performed using Taqman gene expression assays for *Il1β* Mm01336189_m1, *inos* Mm01309893_m1, *Mcp1* Mm00441242_m1, Ifnγ Mm00439552_s1, *Mmp-9* Mm00442991_m1, *Timp-1* Mm00441818_m1, *Loxl-2* Mm00804740_m1, *Col1a1* Mm00801666_g1 and *Gapdh* 4352339E. Relative gene expression was calculated using the ΔΔCt-method.

### Collagen layer thickness measurement

Samples were processed using a benchtop tissue processor (Leica TP 1020) prior to be embedded and cut into 5 µm sections. To visualize the collagen layer, the samples were stained with Sirius Red according to a standard protocol^33^. Sirius red staining was examined using the Imager Z2 microscope (Zeiss) and the software AxioVision (Zeiss). Quantification of the Sirius red-stained collagen was performed by ImageJ 1.47t (NIH, USA) using pictures taken under transmission light, as well as a polarized light filter. Collagen layer thickness was determined in 5 μm sections by a blinded investigator from at least eight places in representative areas at 10-fold magnification. Sirius Red-stained slides were analyzed by bright-field microscopy with an additional polarizing filter. Under polarized light, Sirius Red-stained collagen assumes a palette of colors ranging from green to red based on the fibrotic maturation process. Elastica van Gieson-stained slides were analyzed by transmission light microscopy.

### Statistical analysis

Statistical analysis for qPCR and collagen layer thickness was performed using Kruskal-Wallis one way analysis of variance on ranks, all pairwise multiple comparison procedures, Dunn’s method. Comparison between genotypes was performed using the Mann-Whitney-Test for each time point. Differences were considered significant at a *p*-value of < 0.05 (*) and highly significant at a *p*-value of < 0.01 (**) and *p*-value of < 0.001 (***).

### Ethical considerations

The experimental protocol for animals was approved by the local Animal Care Committee of the University of Zurich (registration numbers 114/2011 and ZH183/2014). We herewith confirm that all experiments were performed in accordance with relevant guidelines and regulations.

## Results

### Fibrogenesis is not affected by inflammatory gene expression levels in the murine model of spontaneous colitis

**Figure 1:**
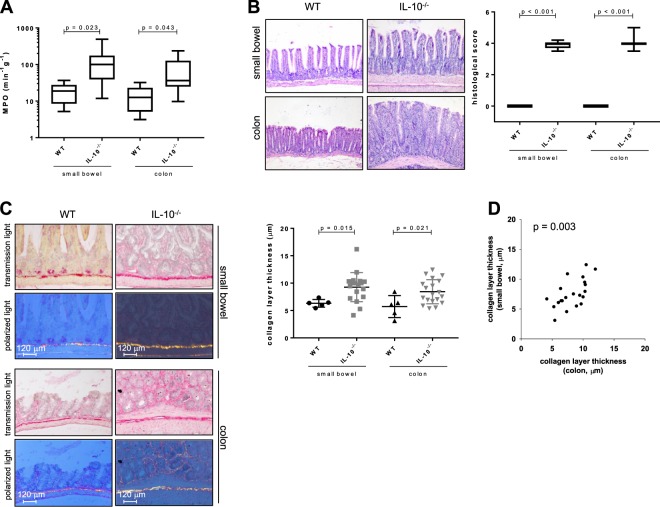
Inflammation is a pre-requisite for the initiation of fibrosis in the spontaneous colitis model. (**A**) MPO. (**B**) Histological score. (**C**) Collagen layer thickness from small bowel and colon. Mann-Whitney rank sum test each. (**D**) Significant link between collagen layer thickness from the small bowel and the colon (Pearson product moment correlation).

We hypothesized that the level of inflammation plays a minor role in fibrosis formation in murine intestine. Three different murine models were used. At first, the *Il10*^−/−^ model of spontaneous colitis was applied. *Il10*^−/−^ mice showed a significantly increased MPO and histological score in the intestinal mucosa compared to WT in both small bowel and colon (Fig. 1A,B). This is reflected by an increased lymphocyte influx into the mucosa (Fig. 1B). Regarding fibrosis parameters, *Il10*^−/−^ mice showed a significantly increased collagen layer thickness compared to WT in both the small bowel and the colon (Fig. 1C). We found a correlation between the collagen layer thickness of the small bowel and the colon (Fig. 1D).

**Figure 2:**
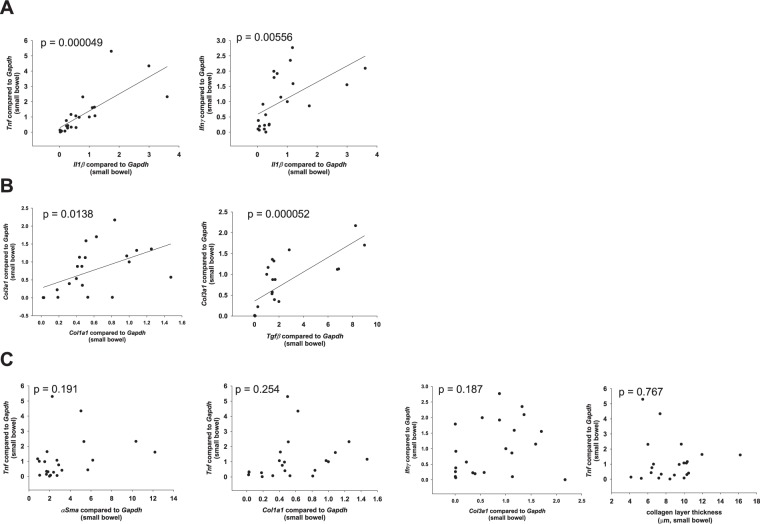
No correlation between pro-inflammatory and pro-fibrotic parameters in the spontaneous colitis model. qPCR from small bowel whole tissue, and collagen layer thickness measurement from the small bowel. (**A**) Pro-inflammatory factors. (**B**) Pro-fibrotic factors. (**C**) Pro-inflammatory parameters do not correlate with pro-fibrotic parameters (Pearson product moment correlation each, nonlinear regression shown if p < 0.05).

**Figure 3:**
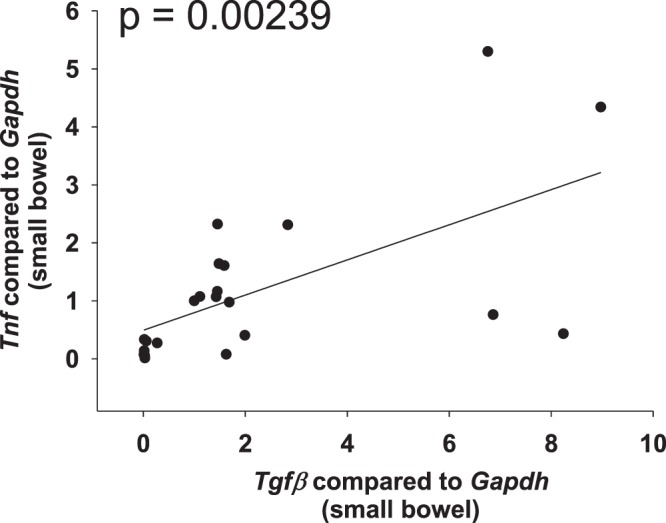
*Tgfβ*, which shares pro-inflammatory and pro-fibrotic features, correlates with pro-inflammatory *Tnf*. qPCR from small bowel whole tissue from mice suffering from spontaneous colitis (Pearson product moment correlation nonlinear regression).

In the small bowel from *Il10*^−/−^ mice, we determined a positive correlation between pro-inflammatory factors. *Il1β* is similarly regulated to *Tnf*, *Ifnγ*, *Mcp1* and *Il6* (Fig. 2A and Table 1, cytokines were assigned to pro-fibrotic or anti-fibrotic function according to Rieder *et al*.^34^). In addition, we determined a positive correlation between pro-fibrotic factors. *Col3a1* is similarly regulated to *Col1a1*, *Tgfβ* and *αSma* (Fig. 2B and Table 1). In contrast, no significant correlation was determined between pro-inflammatory *Tnf* and pro-fibrotic *αSma*, *Col1a1*, *Col3a1*, and collagen layer thickness (Fig. 2C and Table 1). In addition, no significant correlation was determined between the HYP content and the expression levels of pro-inflammatory markers. Likewise, no significant correlation was determined between pro-fibrotic *Col1a1* and pro-inflammatory *Tnf*, *Il1β*, *Ifnγ*, *Il6 or* MPO (Fig. 2C and Table 1). During spontaneous colitis, changes in inflammatory expression were not reflected by changes in fibrosis associated gene expression. *Tgfβ*, which has a dual function sharing pro-inflammatory and pro-fibrotic features, correlated with pro-inflammatory *Tnf* (Fig. 3). qPCR data from colon whole tissue confirmed the results from small bowel (Supplementary Fig. 1).

**Table 1:** No significant correlation between pro-inflammatory *Tnf* and pro-fibrotic markers. qPCR from small bowel whole tissue sections. Pearson product moment correlation. *Tgfβ*, *Mcp1*, *Il1β* and *Ifnγ* were assigned to pro-fibrotic or anti-fibrotic function according to Rieder *et al*.^34^.

			pro-fibrotic							anti-fibrotic
						pro-inflammatory				
			*αSma*	*Col1a1*	*Col3a1*	*Tgfβ*	*Mcp1*	*Il1β*	*Tnf*	*Ifnγ*
pro-fibrotic	pro-inflammatory	***αSma***		0.0176	0.00452	NS	0.00279	0.007	NS	NS
		***Col1a1***	0.0176		0.0138	NS	0.0129	NS	NS	NS
		***Col3a1***	0.00452	0.0138		0.0000518	0.0081	0.00671	NS	NS
		***Tgfβ***	NS	NS	0.0000518		NS	NS	0.00239	NS
		***Mcp1***	0.00279	0.0129	0.0081	NS		0.00524	0.0158	NS
		***Il1β***	0.007	NS	0.00671	NS	0.00524		0.000049	0.00556
		***Tnf***	NS	NS	NS	0.00239	0.0158	0.000049		NS (0.0861)
anti fibrotic		***Ifnγ***	NS	NS	NS	NS	NS	0.00556	NS (0.0861)

### Fibrogenesis is not affected by inflammatory gene expression levels in the DSS-induced model of chronic colitis

**Figure 4:**
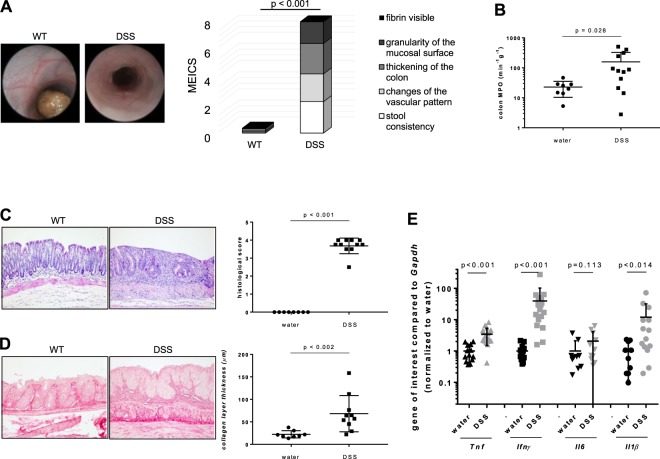
Inflammation is a pre-requisite for the initiation of fibrosis in DSS-induced chronic colitis. (**A**) MEICS. (**B**) MPO. (**C**) Histological score. (**D**) Collagen layer thickness from colon. (**E**) qPCR. Mann-Whitney rank sum test each.

As a second experimental approach we used the DSS-induced chronic colitis model. Regarding inflammatory parameters, mice suffering from chronic colitis showed a significantly increased MEICS compared to WT (Fig. 4A). Mice suffering from chronic colitis showed a significantly increased MPO (Fig. 4B) and histological score in the colon samples (Fig. 4C). This is reflected by an increased lymphocyte influx. Mice suffering from chronic colitis showed a significantly increased collagen layer thickness compared to WT (Fig. 4D). Pro-inflammatory *Tnf*, *Ifnγ*, *Il6* and *Il1β* mRNA expression levels were increased in colitis (Fig. 4E).

**Figure 5:**
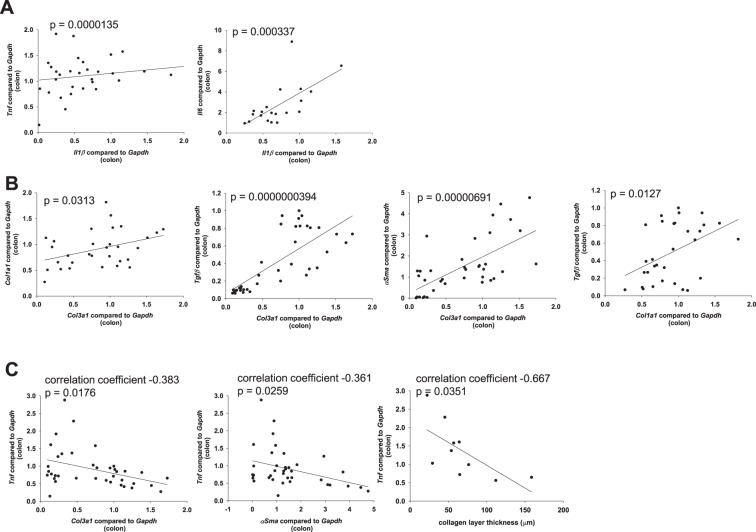
No correlation between pro-inflammatory and pro-fibrotic parameters in DSS-induced chronic colitis. qPCR from colon whole tissue and collagen layer thickness measurement from colon. (**A**) Pro-inflammatory factors. (**B**) Pro-fibrotic factors. (**C**) Pro-inflammatory parameters do not correlate with pro-fibrotic parameters (Pearson product moment correlation each, nonlinear regression shown if p < 0.05).

**Figure 6:**
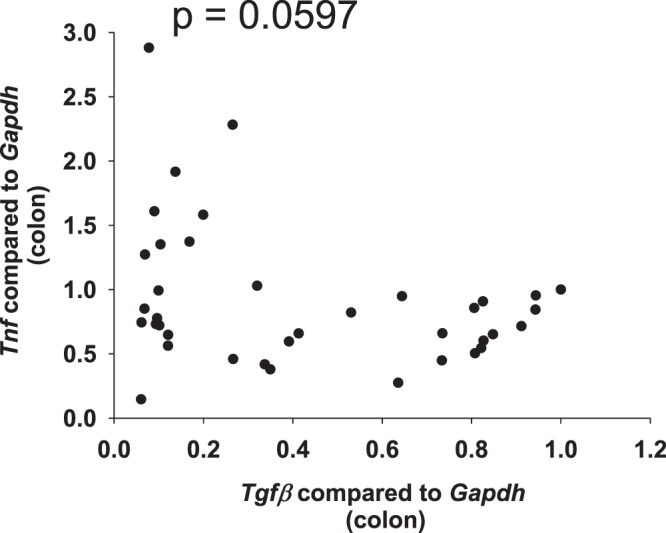
*Tgfβ*, which shares pro-inflammatory and pro-fibrotic features, correlates with pro-inflammatory *Tnf*. qPCR from colon whole tissue from mice suffering from DSS-induced chronic colitis (Pearson product moment correlation nonlinear regression).

In the colon from mice suffering from DSS-induced chronic colitis, we determined a positive correlation between pro-inflammatory factors. *Il1β* is similarly regulated to *Tnf* and *Il6* (Fig. 5A). In addition, we determined a positive correlation between pro-fibrotic factors. *Col3a1* is similarly regulated to *Col1a1*, *Tgfβ* and *αSma* (Fig. 5B). In contrast, no significant correlation was determined between pro-inflammatory *Tnf* and pro-fibrotic *Col3a1*, and collagen layer thickness (Fig. 5C). During DSS-induced chronic colitis, changes in inflammatory expression were not reflected by fibrosis associated gene expression. *Tgfβ*, which exhibits pro-inflammatory and pro-fibrotic features, showed a trend towards a significant correlation to pro-inflammatory *Tnf* (Fig. 6).

### Fibrotic gene expression is not affected by inflammation in the heterotopic transplantation model

In a third experimental approach we performed transplantation of the intestine according to the heterotopic animal model for intestinal fibrosis. For this purpose small bowel resections from both non-inflamed WT and inflamed IL-10 deficient mice were used. IL-10 is a potent macrophage-deactivating cytokine, which is associated with the suppression of IFNγ and iNOS expression.

**Figure 7:**
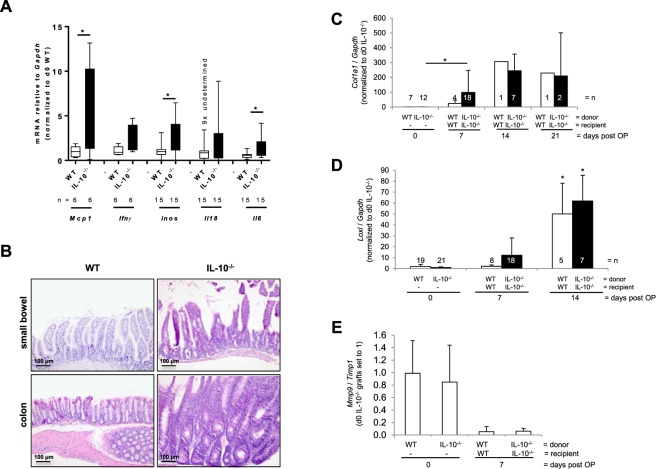
Induction of Col1a1 and Loxl mRNA expression during fibrosis reach the same level in grafts from I*l10*^−/−^ and WT donors in the transplant model of fibrosis. (**A**) qPCR confirmed increase of *Mcp1*, *Ifnγ*, *inos*, *Il18* and *Il6* in the small bowel from *Il10*^−/−^ donor mice compared to WT (*p < 0.05). (**B**) H&E. Lymphocyte influx is increased in small bowel and colon of *Il10*^−/−^ compared to WT. (**C**) *Col1a1* mRNA expression is significantly increased in grafts from *Il10*^−/−^ donor mice transplanted into *Il10*^−/−^ recipients at day seven compared to freshly isolated intestine from *Il10*^−/−^. (**D**) *Loxl* mRNA expression is significantly increased in grafts from both *Il10*^−/−^ donor mice transplanted into *Il10*^−/−^ recipients and WT donor mice transplanted into WT recipients from day 14 compared to freshly isolated intestine and grafts from day 7. (**E**) *Mmp9*/*Timp1 ratio* is decreased in grafts from day 7 compared to freshly isolated intestine independent from the genotype (*p < 0.05, n = as indicated).

Control tissue was obtained from *Il10*^−/−^ and WT mice. Here, mRNA expression of *Mcp1*, *Ifnγ*, *inos*, *Il18 and Il6*. *Mcp1*, *inos* and *Il6* was significantly increased in freshly isolated resections from *Il10*^−/−^ compared to WT mice (Fig. 7A). *Ifnγ* and *Il18* expression further confirmed increased inflammation in *Il10*^−/−^ mice compared to WT mice. Both small bowel and colon showed massive lymphocyte influx into the mucosa in inflamed *Il10*^−/−^ mice compared to non-inflamed WT mice (Fig. 7B).

Following the initiation of fibrosis by heterotopic transplantation, we investigated whether increased inflammation in the absence of IL-10 has an effect on fibrotic processes. 7 days after transplantation, *Col1a1* mRNA expression was significantly increased in grafts from *Il10*^−/−^ donors transplanted into *Il10*^−/−^ recipients compared to freshly isolated small bowel from *Il10*^−/−^ mice (Fig. 7C, 98.3 ± 149.6 vs. 1.0 ± 0.7, respectively, n = as indicated, p < 0.05*). In comparison to grafts from IL-10^−/−^, *Col1a1* expression in grafts from WT donors transplanted into WT recipients appeared to be delayed on day 7 (Fig. 7C, 98.3 ± 149.6 vs. 24.8 ± 21.9, respectively). However, fibrosis was further increased 14 and 21 days after transplantation in a time-dependent manner in grafts from both *Il10*^−/−^ and WT donors showing similar values.

Lysyl oxidase homolog (LOXL) catalyses the first step in the formation of crosslinks in collagens and elastin. *Loxl* mRNA expression in grafts from WT donors appeared to be delayed on day 7 compared to grafts from *Il10*^−/−^ mice (Fig. 7D, 12.2 ± 15.9 vs. 2.2 ± 1.2, respectively). *Loxl* expression was further increased 14 days after transplantation in grafts from both *Il10*^−/−^ and WT donors showing similar values (Fig. 7D, 61.9 ± 23.4 vs. 50.0 ± 28.1, respectively). Although *Loxl* expression was significantly increased in grafts compared to freshly isolated small bowel (day 0, Fig. 7D, *p < 0.05) no difference could be determined between grafts from *Il10*^−/−^ and WT donors suggesting that preceding inflammation of resections has no effect on fibrogenesis.

As genes responsible for assembly of collagen during fibrogenesis were increased in fibrogenesis in a time dependent manner irrespectively of the presence of IL-10, we next determined the expression of matrix metalloproteinase (*Mmp9)* and its natural inhibitor tissue inhibitor of metalloproteinase (*Timp)1*, which is also relevant for disassembly of collagen. Grafts isolated from both *Il10*^−/−^ and WT recipient mice showed a decrease in *Mmp9*/*Timp1* ratio when compared to freshly isolated small bowel. Ratios from both *Il10*^−/−^ and WT recipients showed similar values (Fig. 7E).

### Collagen deposition during fibrogenesis is independent of preceding inflammation

**Figure 8:**
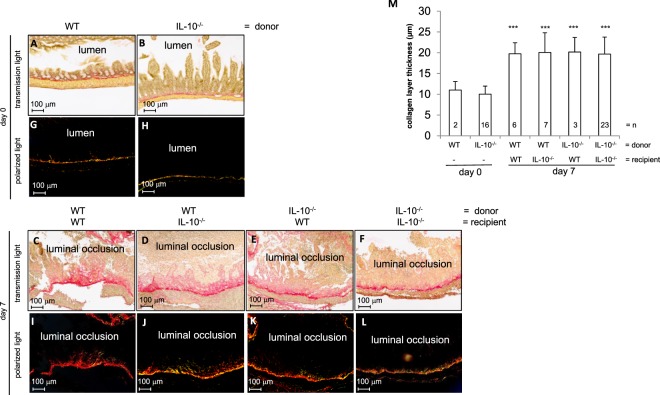
Development of intestinal fibrosis is not prevented in grafts from *Il10*^−/−^ donors in the transplant model of fibrosis. Sirius red staining. Transmission light showed the collagen layer in freshly isolated grafts from (**A**) WT and (**B**) IL-10^−/−^ mice in comparison to an increased collagen layer thickness in grafts at day seven from WT donor mice transplanted into (**C**) WT and (**D**) IL-10^−/−^ recipients next to grafts from IL-10^−/−^ donors transplanted into (**E**) WT and (**F**) IL-10^−/−^ recipients. (**G**,**H**,**I**–**L**) Polarized light. (**M**) Collagen layer thickness increased significantly in grafts following transplantation. Kruskal-Wallis One Way Analysis of Variance on Ranks, All Pairwise Multiple **C**omparison Procedures (Dunn’s Method), mean value, SD (*** p < 0.001).

Next, we determined collagen deposition after heterotopic transplantation in histological cross sections of grafts from *Il10*^−/−^ and WT donors using Sirius red staining under transmission light microscopy (Fig. 8). Freshly isolated small intestine was characterized by an open lumen and distinctive epithelial crypts (Fig. 8A,B). At day 7 after transplantation, the lumen of intestinal grafts was obstructed by granulation tissue and fibrotic material (Fig. 8C–F). Polarizing light microscopy revealed collagen adjacent to the submucosa in freshly isolated small intestine (Fig. 8G,H). In contrast, collagen deposition was increased in the submucosa and in the luminal occlusion following transplantation (Fig. 8I–L). Collagen layer thickness in harvested grafts was significantly increased in comparison to freshly isolated small intestine irrespective of the genotype of donor and recipient mice (Fig. 8M, p < 0.001***), indicating that collagen deposition was unrelated to preceding inflammation. Loss of epithelial structures and luminal occlusion was observed in grafts from both IL-10^−/−^ and WT donors. An increase in collagen deposition independent of preceding inflammation of the grafts was also confirmed by Elastica van Gieson (EvG) staining (Supplementary Fig. 2).

### The expression of pro-inflammatory *Il1β* does not correlate with collagen layer thickness in the heterotopic transplant model

**Figure 9:**
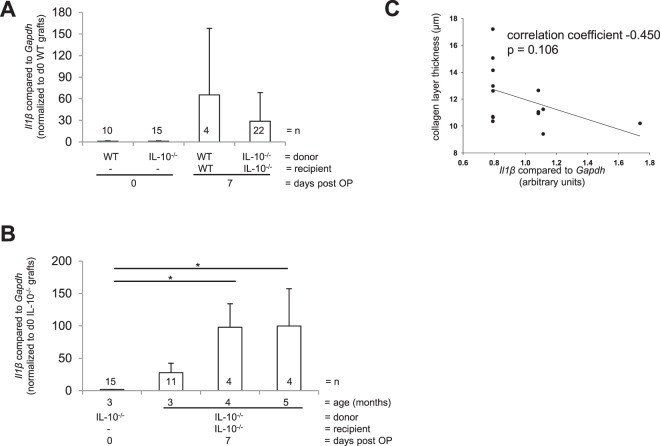
*Il1β* expression does not correlate with collagen layer thickness in the transplant model of fibrosis. (**A**) *Il1β* is increased in grafts from both IL-10^−/−^ donor mice transplanted into IL-10^−/−^ recipients and WT donor mice transplanted into WT recipients from day 7 compared to freshly isolated intestine. **(B)** qPCR confirmed that *Il1β* mRNA expression is significantly increased in grafts from IL-10^−/−^ donor mice four and five months of age transplanted into *Il10*^−/−^ recipients at day 7 compared to freshly isolated intestine from *Il10*^−/−^ (*p < 0.05, n = as indicated). **(C)** *Il1β* does not correlate with collagen layer thickness (Pearson correlation p > 0.050).

Pre-existing intestinal inflammation could have an impact on the severity of inflammatory processes during fibrogenesis. Therefore, we next aimed to determine the severity of inflammation during fibrogenesis in both WT and IL-10^−/−^ mice. *Il1β* expression in grafts was increased 7 days after transplantation compared to freshly isolated small bowel, but grafts of both WT and IL-10^−/−^ donors showed similar values during fibrogenesis (Fig. 9A, 65.4 ± 46.2 and 28.8 ± 8.3 compared to 1.0 ± 0.19 and 1.0 ± 0.26, respectively). IL-10^−/−^ mice develop a chronic colitis after 12–15 weeks that worsens in an age-dependent manner. Therefore, we next analyzed whether mRNA expression of *Il1β* depends on the age of donors. *Il1β* mRNA expression was increased in grafts from three months old IL-10^−/−−/−^ donors transplanted into IL-10^−/−^ recipients compared to freshly isolated small bowel from IL-10^−/−^ mice (Fig. 9B, 27.7 ± 14.6 vs. 1.7 ± 0.3, respectively), and was significantly increased in grafts from both four and five months old IL-10^−/−^ donors compared to freshly isolated small bowel (Fig. 9B, p < 0.05*). However, *Il1β* mRNA expression was not correlated to collagen layer thickness of grafts (Fig. 9C). Moreover, the collagen layer thickness in harvested grafts was not increased in an age dependent manner (Supplementary Fig. 3), suggesting no correlation of fibrogenesis to the inflammatory status of the donor.

**Figure 10:**
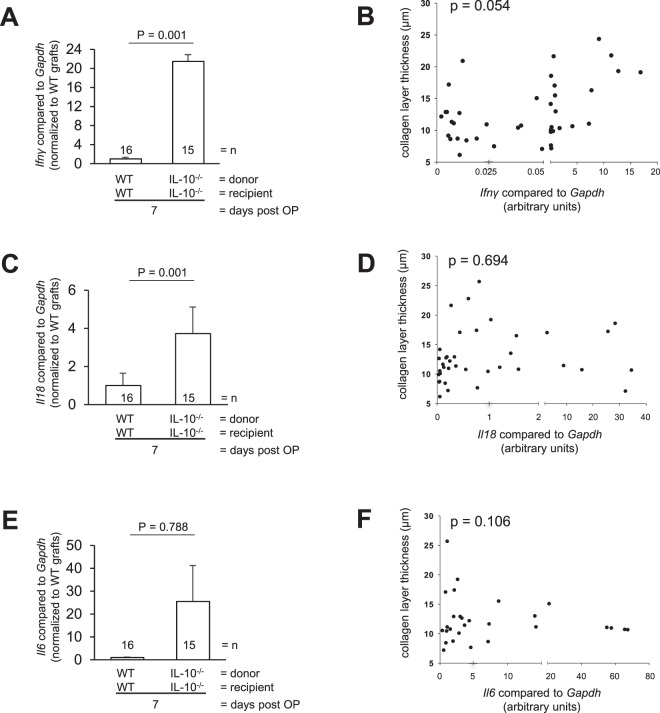
*Ifnγ*, *Il18* and *Il6* expression do not correlate with collagen layer thickness in the transplant model of fibrosis. (**A**) Anti-fibrotic *Ifnγ* is significantly increased in grafts from *Il10*^−/−^ donor mice transplanted into *Il10*^−/−^ recipients from day 7 compared to WT. (**B**) *Ifnγ* does not correlate with collagen layer thickness. (**C**) Pro-inflammatory *Il18* and (**E**) *Il6* is significantly increased in *Il10*^−/−^ compared to WT. (**D**) *Il18* and (**F**) *Il6* do not correlate with collagen layer thickness (Mann-Whitney rank sum test as indicated and Pearson correlation p > 0.050 each).

Further, grafts of *Il10*^−/−^ donors showed increased levels of *Ifnγ*, *Il18* and *Il6* mRNA expression compared to WT (Fig. 10A–C,E), but mRNA expression was not correlated to collagen layer thickness of grafts (Fig. 10B–D,F).

## Discussion

In this study we investigated how the severity of inflammation affects the development and extent of intestinal fibrosis in three different mouse models. Both the *Il10*^−/−^ mouse model and the DSS-induced model of chronic colitis have been proposed as appropriate for investigating intestinal fibrosis because in the chronic phase, an accumulation of ECM is detectable in the affected intestine. In both, the *Il10*^−/−^ model of spontaneous colitis and the DSS-induced model of chronic colitis, fibrosis developed following intestinal inflammation. This is similar to the clinical observation that fibrosis only develops in gut segments where inflammation is or was present^8^. Model specific progressions of the inflammatory status, like the increased expression of pro-inflammatory mRNAs and the increase in MPO, were associated with increased expression of pro-fibrotic mRNAs, deposition of ECM and thickening of the bowel wall. However, pro-inflammatory markers were not correlated to fibrosis associated markers in a significant manner. Deposition of ECM and activation of fibroblasts could be uncoupled from the degree of inflammation in the course of fibrogenesis.

Furthermore, we investigated the link between the severity of the inflammation and the lack of IL-10 on the development of fibrosis using the heterotopic transplantation model. To study the potential impact of the severity of inflammation we studied IL-10 deficient animals as donors and recipients of the graft. Anti-inflammatory IL-10 is a potent macrophage-deactivating cytokine^35,36^ and a suppressor of iNOS expression^37,38^. Conversely, IL-10 depletion results in an increase of IFNγ in colonic mucosa^39^. Although determined pronounced differences in the expression of these cytokines and mediators between IL-10^−/−^ and WT animals, as well as a massive influx of lymphocytes in the mucosa of IL-10^−/−^, the development of intestinal fibrosis was not influenced by the IL-10 deficiency. A very similar development of fibrosis was found in grafts from both IL-10^−/−^ and WT donors. Collagen expression and deposition, which increased over time, was virtually identical in both IL-10^−/−^ and WT grafts after 14 and 21 days. Increased collagen deposition in the intestinal wall was determined by histological analysis, and confirmed the qPCR data. EvG and Sirius red staining demonstrated an identical network of collagen fibrils accumulating progressively in grafts from both genotypes. Expression of genes responsible for assembly of ECM in fibrogenesis, as well as collagen deposition in intestinal grafts showed similar values regardless of the gentic background of donors and recipient and the lack or presence of IL-10. Therefore, we assume that the severity of inflammation during fibrogenesis does not correlate with collagen deposition.

Several animal models for the study of intestinal fibrosis have been proposed^8^. All of them have some advantages as well as disadvantages and none of them really resembles intestinal fibrosis of CD patients. The transplant model of intestinal fibrosis is also an artificial model and therefore may have its own limitations. The exact cause for the initiation and progression of fibrosis in the heterotopic intestinal transplant model is unknown. Graft rejection, ischemia, hypoxia, increased *Hif1α* and neo-vascularisation could contribute to fibrosis in this animal model^32,40^. An excessive synthesis of ECM and activation of intestinal myofibroblasts positive for both vimentin and αSMA, hallmarks in the development of fibrosis, are detectable in the heterotopic transplant animal model of intestinal fibrosis^32,41^. Additionally, IL-13, a known inducer of TGFβ expression, is significantly increased in grafts in a time-dependent manner^32^. The immune response to grafts most probably is strain specific. Previously, we observed an increase in neutrophils in the heterotopic transplantation model that may be affected by the immune response to intestinal grafts, rejection, ischemia or hypoxia, which contributes to fibrosis in the animal model but not necessarily to fibrosis in IBD patients^40^. Differences in the microbiome are likely to influence the development of intestinal fibrosis.

There is already mounting evidence that intestinal fibrogenesis once initiated is no longer depending on the presence of inflammation^14^. For example, in the *S*. *typhimurium* colitis model very early antibiotic intervention with levofloxacin repressed bacteria-induced inflammation and subsequent fibrosis. Late eradication of bacteria did not alter the development of intestinal fibrosis, suggesting that fibrosis is self-propagative. Furthermore, pulmonary fibrosis experiments in mice treated with BLM intratracheally indicate that fibrosis could develop independently from preceding inflammation. Further, fibrosis could be induced in C57BL/6 SCID mice lacking mature T and B cells, suggesting that lymphocytes are not required for the induction of the disease^17,42^. IL-12p40^−/−^ mice lacking a p70 cytokine to promote T-helper-1-mediated inflammation showed less inflammation upon BLM compared to WT, whereas pulmonary fibrosis and hydroxyproline content were increased in IL-12p40^−/−^ mice^16^. The pro-fibrotic cytokine IL-6, capable of fibroblast activation and promotion of collagen synthesis, was found to be substantially increased in IL-12p40^−/−^ mice, and was suggested to promote BLM-mediated lung fibrosis.

The importance of IL-10-dependent signalling is well established, as well as the impact of IL-10 deficiency in the initiation of inflammation in different animal models and diseases. Nonetheless, the exact role of IL-10 in the subsequent development of fibrosis remains unclear and is subject of some discussion. In BLM-mediated injury, pulmonary inflammation was promoted in IL-10^−/−^ mice, as reflected by an increased representation of lymphocytes compared to WT^15^, but no difference was found in the degree of pulmonary fibrosis, as evidenced by histology and hydroxyproline content. These studies suggest an immunomodulary role for IL-10^−/−^ in the inflammatory response but not in BLM-induced pulmonary fibrosis and are in line with our findings in the heterotopic transplant model of small intestinal fibrosis.

On the other hand, there is evidence from many different animal models that lack of IL-10 may lead to fibrosis, and administration of IL-10 is followed by decreased fibrosis. However, studies on the distinction between the role of IL-10 in the initiation of inflammatory processes and the development of fibrosis are limited. Recent data show that IL-10 may display anti-fibrotic effects during unilateral ureteral obstruction through surgical ligation^43^. Histological analysis showed an increased rate of tubular injury in IL-10 deficient mice. Collagen deposition and activation of myofibroblasts were also increased compared to WT mice. Interestingly, the expression pro-inflammatory IL-6 was increased in IL-10 deficient mice, suggesting a crucial role for IL-10-induced IL-6 in the protection from renal fibrosis. In a model of granulomatous experimental thyroiditis, lack of IL-10 led to ongoing inflammation and fibrosis associated with the destruction of thyroid follicles. In this model, IL-10 is required to activate FasL on T cells initiating apoptosis^44^, therefore, the absence of IL-10 increases the lifespan of activated lymphocytes and maintains inflammation. In a model of cerulean-induced acute pancreatitis, a severe increase of fibrosis was observed in IL-10 knockout mice compared to WT^45^, together with increased TGF-β1 plasma levels in pancreatic cells. Histological evaluation of collagen deposition in the pancreas of IL-10 deficient mice confirmed increased fibrogenesis compared to WT. Recently, Mentink-Kane *et al*. also demonstrated the anti-fibrotic properties of IL-10 in a pathogen driven inflammation model of the liver^46^. These authors showed that IL-10, IL-12p40 and IL-13Ralpha act cooperatively through reduction of pro-fibrotic IL-13. A positive effect of treatment with recombinant IL-10 was shown in a rat model with CCL4 induced hepatic fibrosis^47^. Upon administration of recombinant IL-10 animals showed a decreased collagen deposition in the fibrotic liver.

Our data suggest that, although IL-10 reduces inflammation, fibrogenesis is uncoupled from IL-10 signalling. The degree of inflammation and the expression levels of pro-inflammatory cytokines had no impact on the development of fibrosis in our model. From a clinical perspective, current anti-inflammatory agents effectively treat inflammatory flares in IBD patients, but at present, there is no specific treatment option for patients with recurrent intestinal fibrosis^48,49^. In consequence, the incidence of strictures and subsequent surgical interventions remains relatively unchanged^50^. Treatment avenues outside the classical anti-inflammatory approaches might end up more promising.

## Electronic supplementary material

supplementary figures 1–3

## References

[1] Rogler G, Zeitz J, Biedermann L (2016). The Search for Causative Environmental Factors in Inflammatory Bowel Disease. Digestive diseases (Basel, Switzerland).

[2] Ina, K. *et al*. Resistance of Crohn’s disease T cells to multiple apoptotic signals is associated with a Bcl-2/Bax mucosal imbalance. *Journal of immunology (Baltimore*, *Md*.*: 1950*) **163**, 1081–1090, doi:ji_v163n2p1081 [pii] (1999). 10395708

[3] Rieder F, Brenmoehl J, Leeb S, Scholmerich J, Rogler G (2007). Wound healing and fibrosis in intestinal disease. Gut.

[4] Paul G, Khare V, Gasche C (2012). Inflamed gut mucosa: downstream of interleukin-10. Eur J Clin Invest.

[5] Murai M (2009). Interleukin 10 acts on regulatory T cells to maintain expression of the transcription factor Foxp3 and suppressive function in mice with colitis. Nat Immunol.

[6] Jones MK, Tomikawa M, Mohajer B, Tarnawski AS (1999). Gastrointestinal mucosal regeneration: role of growth factors. Frontiers in bioscience: a journal and virtual library.

[7] Sartor RB (1995). Current concepts of the etiology and pathogenesis of ulcerative colitis and Crohn’s disease. Gastroenterology clinics of North America.

[8] Rieder F, Fiocchi C, Rogler G (2017). Mechanisms, Management, and Treatment of Fibrosis in Patients With Inflammatory Bowel Diseases. Gastroenterology.

[9] Cosnes J (2002). Long-term evolution of disease behavior of Crohn’s disease. Inflammatory bowel diseases.

[10] Louis E (2001). Behaviour of Crohn’s disease according to the Vienna classification: changing pattern over the course of the disease. Gut.

[11] Freeman HJ (2003). Natural history and clinical behavior of Crohn’s disease extending beyond two decades. Journal of clinical gastroenterology.

[12] Gordon IO, Agrawal N, Goldblum JR, Fiocchi C, Rieder F (2014). Fibrosis in ulcerative colitis: mechanisms, features, and consequences of a neglected problem. Inflammatory bowel diseases.

[13] Agrawal N (2016). In Ulcerative Colitis Is Linked With Severity and Chronicity of Inflammation. Gastroenterology.

[14] Johnson LA (2012). Intestinal fibrosis is reduced by early elimination of inflammation in a mouse model of IBD: impact of a “Top-Down” approach to intestinal fibrosis in mice. Inflammatory bowel diseases.

[15] Kradin RL (2004). IL-10 inhibits inflammation but does not affect fibrosis in the pulmonary response to bleomycin. Experimental and molecular pathology.

[16] Sakamoto H, Zhao LH, Jain F, Kradin R (2002). IL-12p40(−/−) mice treated with intratracheal bleomycin exhibit decreased pulmonary inflammation and increased fibrosis. Experimental and molecular pathology.

[17] Zhu J, Cohen DA, Goud SN, Kaplan AM (1996). Contribution of T lymphocytes to the development of bleomycin-induced pulmonary fibrosis. Annals of the New York Academy of Sciences.

[18] Ng TH (2013). Regulation of adaptive immunity; the role of interleukin-10. Front Immunol.

[19] Glocker EO, Kotlarz D, Klein C, Shah N, Grimbacher B (2011). IL-10 and IL-10 receptor defects in humans. Annals of the New York Academy of Sciences.

[20] Chaudhry A (2011). Interleukin-10 signaling in regulatory T cells is required for suppression of Th17 cell-mediated inflammation. Immunity.

[21] Sakaguchi S, Miyara M, Costantino CM, Hafler DA (2010). FOXP3+ regulatory T cells in the human immune system. Nat Rev Immunol.

[22] Glocker EO (2009). Inflammatory bowel disease and mutations affecting the interleukin-10 receptor. The New England journal of medicine.

[23] Glocker EO (2010). Infant colitis–it’s in the genes. Lancet.

[24] Franke A (2008). Sequence variants in IL10, ARPC2 and multiple other loci contribute to ulcerative colitis susceptibility. Nature genetics.

[25] van der Linde K (2003). A Gly15Arg mutation in the interleukin-10 gene reduces secretion of interleukin-10 in Crohn disease. Scandinavian journal of gastroenterology.

[26] Noguchi E, Homma Y, Kang X, Netea MG, Ma X (2009). A Crohn’s disease-associated NOD2 mutation suppresses transcription of human IL10 by inhibiting activity of the nuclear ribonucleoprotein hnRNP-A1. Nature immunology.

[27] Ra HJ, Parks WC (2007). Control of matrix metalloproteinase catalytic activity. Matrix biology: journal of the International Society for Matrix Biology.

[28] Agrez M (1999). The alpha v beta 6 integrin induces gelatinase B secretion in colon cancer cells. International journal of cancer.

[29] Agrez MV (1996). Multiplicity of fibronectin-binding alpha V integrin receptors in colorectal cancer. British journal of cancer.

[30] Goffin L (2016). Anti-MMP-9 Antibody: A Promising Therapeutic Strategy for Treatment of Inflammatory Bowel Disease Complications with Fibrosis. Inflammatory bowel diseases.

[31] Obermeier F (1999). Interferon-gamma (IFN-gamma)- and tumour necrosis factor (TNF)-induced nitric oxide as toxic effector molecule in chronic dextran sulphate sodium (DSS)-induced colitis in mice. Clin Exp Immunol.

[32] Hausmann M (2013). A new heterotopic transplant animal model of intestinal fibrosis. Inflammatory bowel diseases.

[33] Rittie L (2017). Method for Picrosirius Red-Polarization Detection of Collagen Fibers in Tissue Sections. Methods in molecular biology (Clifton, N.J.).

[34] Rieder F, Fiocchi C (2009). Intestinal fibrosis in IBD–a dynamic, multifactorial process. Nat Rev Gastroenterol Hepatol.

[35] Fiorentino DF, Zlotnik A, Mosmann TR, Howard M, O’Garra A (1991). IL-10 inhibits cytokine production by activated macrophages. Journal of immunology.

[36] Bogdan C, Vodovotz Y, Nathan C (1991). Macrophage deactivation by interleukin 10. The Journal of experimental medicine.

[37] Becherel PA (1995). Interleukin-10 inhibits IgE-mediated nitric oxide synthase induction and cytokine synthesis in normal human keratinocytes. European journal of immunology.

[38] Seyler I, Appel M, Devissaguet JP, Legrand P, Barratt G (1997). Modulation of nitric oxide production in RAW 264.7 cells by transforming growth factor-beta and interleukin-10: differential effects on free and encapsulated immunomodulator. Journal of leukocyte biology.

[39] Jarry, A., Bossard, C., Sarrabayrouse, G., Mosnier, J. F. & Laboisse, C. L. Loss of interleukin-10 or transforming growth factor beta signaling in the human colon initiates a T-helper 1 response via distinct pathways. *Gastroenterology* **141**, 1887-1896.e1881-1882, doi:10.1053/j.gastro.2011.08.002 (2011). 10.1053/j.gastro.2011.08.002 21839042

[40] Lutz C (2017). Myeloid differentiation primary response gene (MyD) 88 signalling is not essential for intestinal fibrosis development. Sci Rep.

[41] Meier R (2016). Decreased Fibrogenesis After Treatment with Pirfenidone in a Newly Developed Mouse Model of Intestinal Fibrosis. Inflammatory bowel diseases.

[42] Helene M (1999). T cell independence of bleomycin-induced pulmonary fibrosis. Journal of leukocyte biology.

[43] Jin Y (2013). Interleukin-10 deficiency aggravates kidney inflammation and fibrosis in the unilateral ureteral obstruction mouse model. Lab Invest.

[44] Fang Y, Sharp GC, Braley-Mullen H (2008). Interleukin-10 promotes resolution of granulomatous experimental autoimmune thyroiditis. Am J Pathol.

[45] Demols A (2002). Endogenous interleukin-10 modulates fibrosis and regeneration in experimental chronic pancreatitis. Am J Physiol Gastrointest Liver Physiol.

[46] Mentink-Kane MM (2011). Accelerated and progressive and lethal liver fibrosis in mice that lack interleukin (IL)-10, IL-12p40, and IL-13Ralpha2. Gastroenterology.

[47] Huang YH (2006). Therapeutic effect of interleukin-10 on CCl4-induced hepatic fibrosis in rats. World J Gastroenterol.

[48] D’Haens G, Geboes K, Rutgeerts P (1999). Endoscopic and histologic healing of Crohn’s (ileo-) colitis with azathioprine. Gastrointestinal endoscopy.

[49] Vermeire S, van Assche G, Rutgeerts P (2007). Review article: Altering the natural history of Crohn’s disease–evidence for and against current therapies. Alimentary pharmacology & therapeutics.

[50] Solberg IC (2007). Clinical course in Crohn’s disease: results of a Norwegian population-based ten-year follow-up study. Clinical gastroenterology and hepatology: the official clinical practice journal of the American Gastroenterological Association.

