# Supplementary material for: Severity of local inflammation does not impact development of fibrosis in mouse models of intestinal fibrosis
Source: Sci Rep. 2018 Oct 12;8:15182. doi: 10.1038/s41598-018-33452-5 (PMC6185984; doi:10.1038/s41598-018-33452-5)
Supplement: Supplementary file 1 — supplementary figures 1–3 [file 41598_2018_33452_MOESM1_ESM.pdf]

Supplementary figures

Severity of local inflammation does not impact development of fibrosis in mouse models of intestinal fibrosis

Hünerwadel A[1], Fagagnini S[1], Rogler G[1], Lutz C[1], Jaeger S U[23], Mamie C[1], Weder B[1], Ruiz P A[1], Hausmann M[1]

[1] Department of Gastroenterology and Hepatology, University of Zurich, Zurich, Switzerland
[2] Dr. Margarete Fischer-Bosch Institute of Clinical Pharmacology, Stuttgart, Germany
[3] University of Tübingen, Tübingen, Germany

Supplementary figure 1

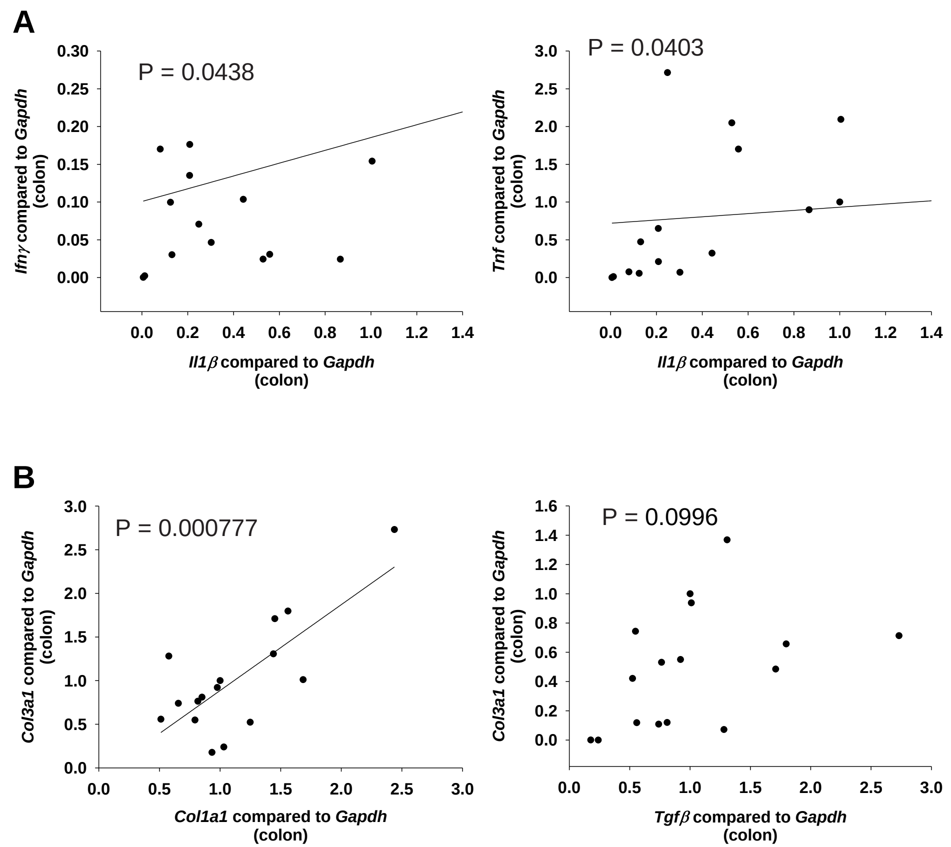

Supplementary figure 1: **No correlation between pro-inflammatory and pro-fibrotic parameters in spontaneous colitis.** qPCR from colon whole tissue and collagen layer thickness measurement from colon. (A) Pro-inflammatory factors. (B) Pro-fibrotic factors. (C) Pro-inflammatory parameters do not correlate with pro-fibrotic parameters (Pearson product moment correlation each, nonlinear regression).

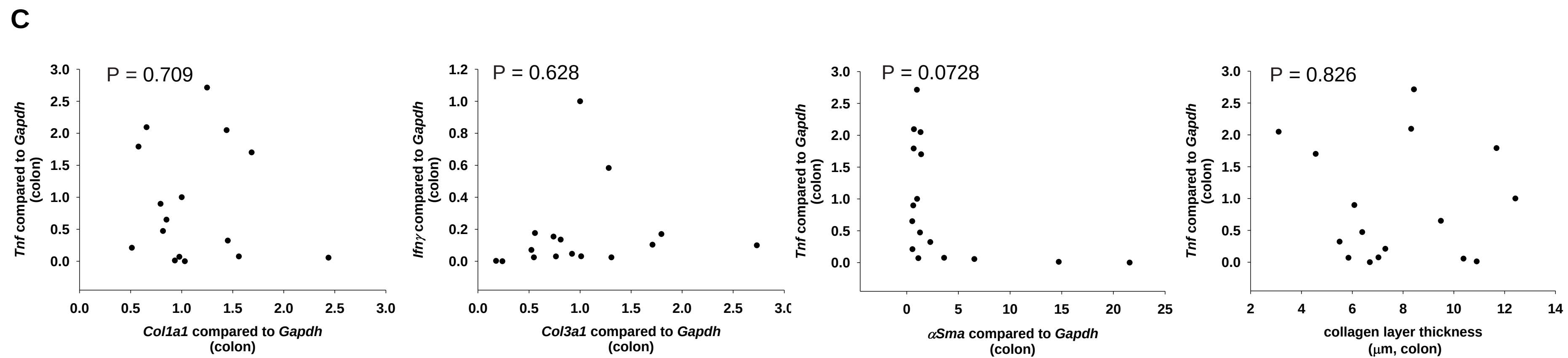

Supplementary figure 2

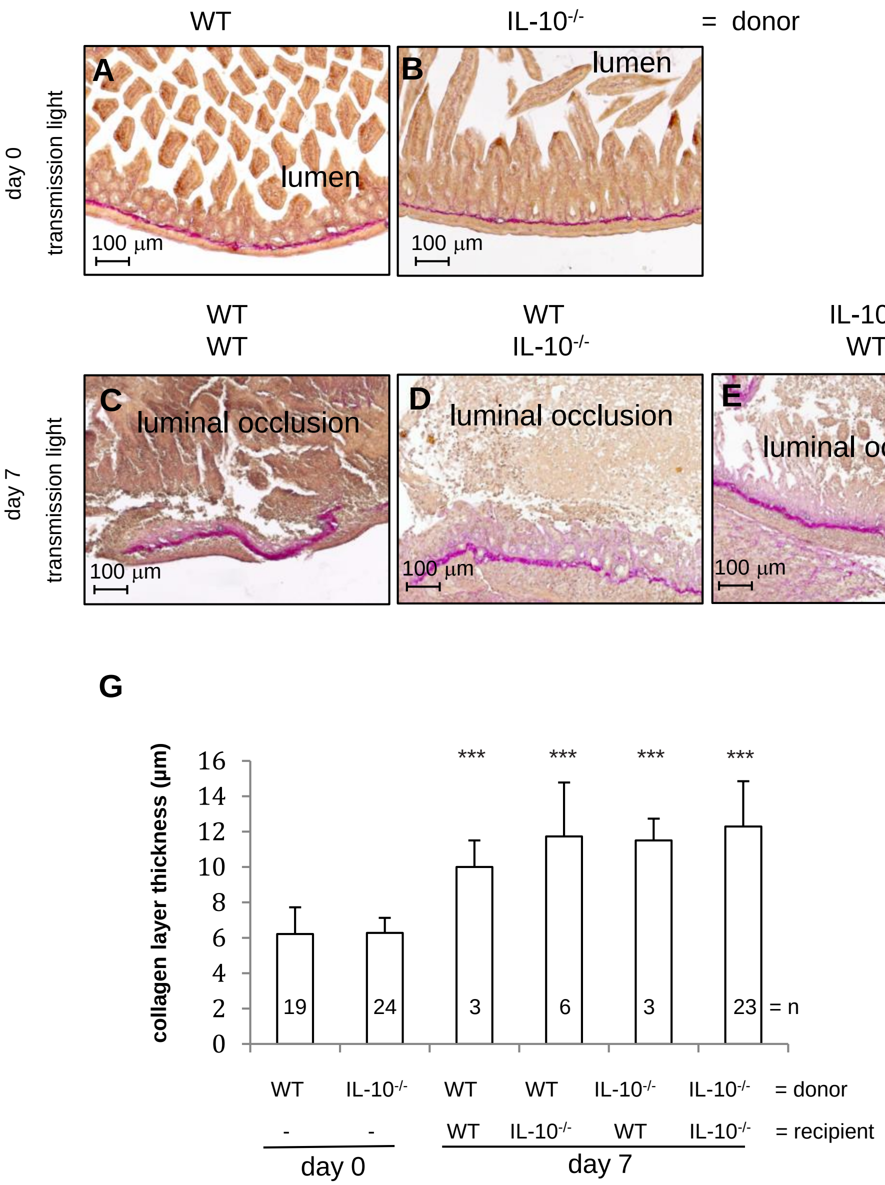

Supplementary figure 2: **Development of intestinal fibrosis is not increased in grafts from IL-10$^{-/-}$ donors compared to WT. (A - F)** EvG staining, transmission light microscopy. **(G)** Thickness was calculated using polarized light microscopy from at least eight places in representative areas at 10-fold magnification for each single graft. Collagen layer thickness was significantly increased in grafts following transplantation. One Way Analysis of Variance, Kruskal-Wallis One Way Analysis of Variance on Ranks, All Pairwise Multiple Comparison Procedures (Dunn's Method), mean value and SD is shown (*** $p < 0.001$, n = as indicated).

Supplementary figure 3

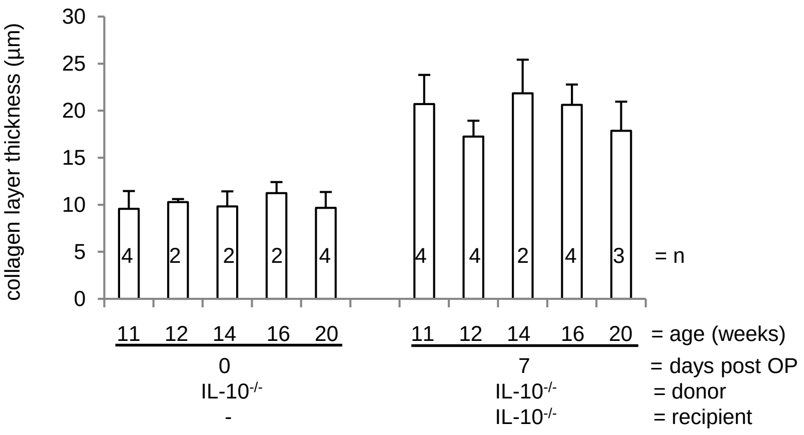

Supplementary figure 3: **Collagen layer thickness in harvested grafts is independent from the age of IL-10$^{-/-}$ donor mice.**
